# Accelerated Anodal tDCS over Right Inferior Frontal Gyrus Improves Inhibitory Control Across Repeated Sessions: Evidence of a Cumulative After Effect

**DOI:** 10.3390/biomedicines14071417

**Published:** 2026-06-23

**Authors:** Daniele Saccenti, Andrea Stefano Moro, Nicolò Geminian, Cecilia Orsi, Jacopo Cappella, Jacopo Lamanna, Mattia Ferro

**Affiliations:** 1 Department of Psychology, Sigmund Freud University, 20143 Milan, Italy; d.saccenti@milano-sfu.it (D.S.); a.moro@milano-sfu.it (A.S.M.); 2 Brain and Behaviour SFU Lab, Sigmund Freud University, 20143 Milan, Italy; 3 Center for Behavioral Neuroscience and Communication (BNC), Vita-Salute San Raffaele University, 20132 Milan, Italy; n.geminian@studenti.unisr.it (N.G.); c.orsi3@studenti.unisr.it (C.O.); j.cappella@studenti.unisr.it (J.C.); 4 Faculty of Psychology, Vita-Salute San Raffaele University, 20132 Milan, Italy

**Keywords:** transcranial direct current stimulation, tDCS, accelerated protocol, right inferior frontal gyrus, rIFG, inhibitory control, response inhibition

## Abstract

**Background/Objectives**: The right inferior frontal gyrus (rIFG) is a key node of the neural network underlying inhibitory control and a promising target for non-invasive brain stimulation. Here, we investigated whether an accelerated anodal transcranial direct current stimulation (tDCS) protocol over the rIFG could produce cumulative improvements in response inhibition. This issue is particularly relevant because cumulative effects of accelerated stimulation cannot be taken for granted, and repeated administrations may lead to progressive improvement, saturation, or no measurable offline persistence. In addition, sham-controlled accelerated studies remain limited. **Methods**: Twenty-two healthy participants underwent three stimulation sessions within the same day. Inhibitory control was assessed using the Stop-Signal task, while Stop-Signal Reaction Time (SSRT) and mean error rate were analyzed by means of linear mixed-effects models. **Results**: Results showed a significant effect of time and a significant stimulation-by-time interaction across the three sessions, indicating a progressive reduction in SSRT during the accelerated protocol. Thus, inhibitory control performance improved as stimulation sessions increased. **Conclusions**: These findings suggest that accelerated anodal tDCS over the rIFG can induce cumulative short-term improvements in inhibitory control. The results support the relevance of the rIFG as a neuromodulation target, while highlighting the importance of interindividual variability and the need for more translationally oriented protocols.

## 1. Introduction

The development of increasingly targeted interventions has become a major clinical and scientific priority. Indeed, standardized protocols do not always guarantee optimal therapeutic outcomes, particularly in mental health, where the multifactorial and biopsychosocial nature of psychopathology calls for treatment approaches capable of addressing a high degree of complexity. In this context, a promising strategy is to focus on transdiagnostic processes, that is, mechanisms impaired across different psychiatric conditions and therefore likely to represent valuable targets for intervention [1]. Among these processes, inhibitory control has received growing attention, as it is considered a core component of executive functioning that enables individuals to regulate behavior by suppressing automatic or prepotent responses when they are inappropriate or counterproductive [2,3]. Inhibitory control encompasses at least two major components: attentional inhibition, which allows irrelevant stimuli to be filtered out, and response inhibition, which suppresses impulsive or maladaptive motor actions [4,5]. In turn, response inhibition can be further divided into reactive inhibition, namely the rapid cancellation of an action in response to an external stop cue, and proactive inhibition, which refers to the anticipatory adjustment of behavior when stopping may be required [6]. These processes are commonly assessed through paradigms such as the Stop-Signal task and the Go/NoGo task, both of which require participants to override prepotent responses under specific conditions [7,8].

Rather than relying on a single “inhibitory center”, inhibitory control depends on a distributed neural network involving both cortical and subcortical regions. Within this network, the right inferior frontal gyrus (rIFG) has been consistently implicated in response inhibition [9,10], together with regions such as the pre-supplementary motor area, anterior cingulate cortex, basal ganglia, and subthalamic nucleus [11,12,13,14]. Dysfunction within this circuitry has been linked to impaired impulse control and maladaptive decision-making across several psychiatric conditions, including attention-deficit/hyperactivity disorder, obsessive–compulsive disorder, substance addiction, and mood disorders [15,16,17,18,19,20,21]. Taken together, these findings support the view that inhibitory control represents a clinically relevant transdiagnostic process and a potentially important target for intervention.

Within this framework, non-invasive brain stimulation (NIBS) offers a promising approach for both investigating and modulating inhibitory-control mechanisms. In particular, transcranial direct current stimulation (tDCS) modulates cortical excitability in a polarity-dependent manner, with anodal stimulation generally associated with increased excitability and cathodal stimulation with decreased excitability [22,23,24]. Although its effects may vary depending on stimulation parameters and interindividual variability [25,26], tDCS remains especially attractive because of its tolerability, relative ease of application, low cost, and potential to induce neuroplastic changes [27,28]. Consistent with this perspective, several studies have shown that anodal tDCS over the rIFG can facilitate inhibitory control, including both reactive and proactive response inhibition [29,30,31], although findings across studies remain partly inconsistent [32,33]. Recent evidence from our group further supports the role of the rIFG as a promising target for neuromodulation. Specifically, we observed that anodal tDCS over the rIFG exerts a general facilitatory effect on inhibitory control, with the clearest effects emerging when performance was assessed with the Stop-Signal task. Under active stimulation, participants showed a significant reduction in Stop-Signal Reaction Time, indicating improved response inhibition. By contrast, effects on the Go/NoGo task were less consistent: anodal stimulation did not significantly affect false alarm rate or Go-trial reaction times, although a significant increase in overall accuracy was observed. These findings suggest that the behavioral effects of rIFG stimulation may depend, at least in part, on the specific inhibitory-control measure employed, with the Stop-Signal task appearing more sensitive than the Go/NoGo task to neuromodulatory effects on response inhibition [34].

At the same time, inhibitory control does not operate in isolation, but interacts with broader higher-order cognitive processes. Among these, delay discounting, defined as the tendency to devalue rewards as the delay to their receipt increases [35], and metacognition, namely the ability to monitor and regulate one’s own cognitive processes [36], may play an important role in shaping inhibitory performance and modulating the effects of neuromodulation. Because both functions rely on prefrontal control mechanisms, they are likely to interact with inhibitory control at both neural and cognitive-behavioral levels [37,38,39,40]. This perspective is particularly relevant in psychiatry, where impairments in inhibitory control, metacognition, and impulsive decision-making frequently co-occur and may jointly contribute to maladaptive behaviors. In line with this view, in our previous study the effects of tDCS on inhibitory control were modulated by individual differences in baseline metacognitive beliefs and delay discounting [34], suggesting that responsiveness to neuromodulation may vary according to the individual’s cognitive profile. Altogether, these findings reinforce the relevance of the rIFG as a target for interventions aimed at enhancing inhibitory control, while also highlighting the need to consider both task characteristics and individual cognitive differences when interpreting tDCS effects.

Building on these findings, the present study aimed to test whether the effects of an accelerated tDCS protocol targeting the rIFG extend beyond the stimulation window and remain detectable during a subsequent short-term offline period. Although the above-mentioned previous evidence indicated that rIFG stimulation enhances inhibitory control, the temporal stability of such an effect remains unknown. Specifically, the current study examines whether the proposed accelerated intervention leaves measurable traces on behavioral performance shortly after the end of stimulation.

This question is particularly relevant because the effects of accelerated stimulation protocols cannot be taken for granted: it remains unclear whether repeated administrations produce a cumulative benefit, whether such effects may instead reach saturation, and whether any gain can be maintained offline after the end of stimulation. In this respect, the accelerated approach adopted here is of particular interest, especially given the relative scarcity of controlled studies employing accelerated neuromodulation protocols with appropriate sham comparison groups. Accordingly, the above-mentioned tDCS studies targeting the rIFG have employed single-session rather than accelerated stimulation protocols. Moreover, the majority of previous investigations testing the effects of accelerated tDCS have targeted other cortical regions (e.g., the dorsolateral prefrontal cortex) and consisted mainly of case reports conducted in psychiatric patients resistant to conventional treatments, which nevertheless suggested potential clinical improvements [41,42,43,44]. Evidence from larger samples of healthy participants remains scarce, with only a single study reporting cognitive benefits following accelerated left temporal lobe stimulation, specifically improvements in executive functioning but not in verbal memory [45]. However, the extent to which these effects are consistently observed across studies remains a matter of ongoing debate, as does the question of whether intensive treatment schedules can affect other cognitive domains and how long such effects might last. Addressing these issues is therefore crucial for evaluating the translational relevance of the protocol, as evidence of cumulative and sustained post-stimulation effects would represent an important step toward the development of rehabilitation-oriented interventions targeting inhibitory control.

## 2. Materials and Methods

### 2.1. Participants

A total of 22 healthy subjects (10 males, 12 females; mean age: 22.59 ± 1.57 years) took part in the study. Most of the participants (*n* = 11) held a high school diploma, whereas the remaining ones possessed either a bachelor’s degree (*n* = 9) or a master’s degree (*n* = 2). Participants were recruited through word-of-mouth basis at a university site and were eligible for inclusion if they had no neurological disorders and no contraindications to non-invasive brain stimulation. In particular, exclusion criteria were as follows: (1) presence of any metallic or electronic implants in the brain/skull (e.g., splinters, fragments, clips, cochlear implants), (2) presence of any metallic or electronic devices at other sites of the body (e.g., cardiac pacemaker, defibrillators or traumatic metallic residual fragments), (3) previous surgical procedures involving the brain or spinal cord, (4) previous trauma-related brain injury followed by impairment of consciousness, (5) previous episodes of fainting spells or syncope, (6) history of skin diseases (e.g., dermatitis, psoriasis or eczema), (7) history of epileptic seizures, (8) being currently pregnant or lactating [46]. All participants were naïve to the aim of the study and provided written informed consent. The study adhered to the ethical principles outlined in the Declaration of Helsinki and received approval from the Ethics Board of the Faculty of Psychotherapy Science and the Faculty of Psychology at Sigmund Freud University (protocol number: XD2735MWC1624L90702).

### 2.2. Transcranial Direct Current Stimulation

**Figure 1:**
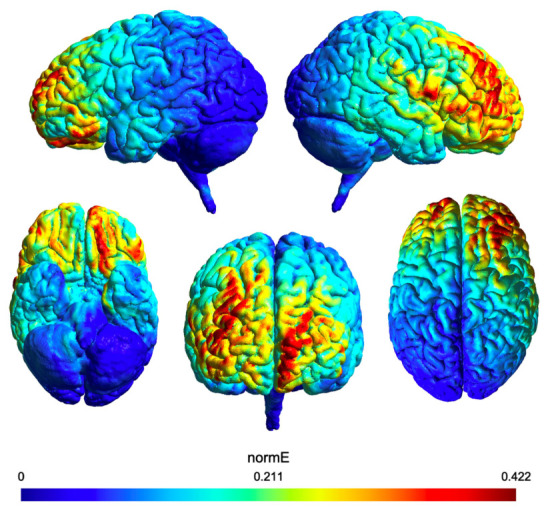
Simulation of the electrical field distribution generated by tDCS stimulation over the rIFG. SimNIBS 4.1.0 (https://simnibs.github.io/simnibs/build/html/index.html, accessed on 1 February 2025) was used in order to estimate the normalized electric field (normE) induced by tDCS [53]. The anode was placed over the crossing point between T4–Fz and F8–Cz, whereas the cathode was positioned over Fp1, according to the 10–20 system.

The tDCS current stimulus was delivered using a battery-driven BrainSTIM device (E.M.S. s.r.l., Bologna, Italy) through a pair of 5 × 5 cm^2^ electrodes inserted in separate saline-soaked sponge bags. Current was applied at 2 mA constant intensity, yielding a current density of 0.08 mA/cm^2^ throughout the entire stimulation, in accordance with safety parameters established for healthy subjects [47,48]. To stimulate the rIFG, the anodal electrode was placed over the crossing point between T4–Fz and F8–Cz, whereas the cathode was positioned over Fp1 (see Figure 1), according to the 10–20 EEG system (see Ditye et al. [49], Jacobson et al. [31], Sandrini et al. [50], and Stramaccia et al. [51] for previous investigations using this montage). The stimulation montage and parameters were the same as those adopted in the reference study [34]. The present study differed from the reference protocol in that stimulation was administered according to an accelerated schedule, with three stimulation blocks delivered within the same day. Each active stimulation block lasted 20 min and included two ramping periods of 15 s at the beginning and end of stimulation. In the sham condition, current was delivered only during brief transient periods at the beginning and end of the session to reproduce the somatosensory sensations of active stimulation [52]. The sham protocol consisted of a 15 s ramp-up, 15 s of stimulation at 2 mA, and a 15 s ramp-down, followed by 18 min and 30 s without stimulation, and ending with the same 45 s sequence. None of the participants reported relevant somatosensory differences between the two conditions, as assessed through a verbal debriefing.

### 2.3. Cognitive Task

Inhibitory control was assessed through a Stop-Signal task (SST) developed in PsyToolkit (Version 3.6.2) [54]. In the SST, participants completed 132 trials, including 99 Go and 33 Stop trials. In Go trials, an arrow pointing either left or right appeared at the center of the screen, and participants were instructed to respond as quickly and accurately as possible by pressing the corresponding left (“B”) or right (“N”) button within 500 ms. If no response was made within this time window, feedback indicating the correct response was provided. In 25% of the trials (Stop trials), a stop signal (a red circle) was presented shortly after the onset of the arrow. Upon presentation of the stop signal, participants were required to withhold their response. The stop signal delay (SSD) was initially randomized between 100 and 450 ms and then adjusted by means of a tracking procedure to maintain an inhibition rate of approximately 50%. The SSD was increased by 50 ms following successful response inhibition and decreased by 50 ms when inhibition failed. The experiment followed a structured trial sequence with an inter-trial interval of 750 ms.

### 2.4. Psychological Measures

Each participant underwent paper-and-pencil testing with the Italian versions of the Metacognitions Questionnaire-30 (MCQ_30_) [55], the Metacognitive Self-Assessment Scale-18 (MSAS_18_) [56], and the Barratt Impulsiveness Scale-11 (BIS_11_) [57]. The MCQ_30_ assesses a range of metacognitive beliefs and processes relevant to the vulnerability and maintenance of psychological disorders across five domains (i.e., cognitive confidence, cognitive self-consciousness, positive beliefs about worry, negative beliefs about worry, and need to control thoughts); items are rated on a 4-point Likert-type scale, with higher scores indicating greater endorsement of dysfunctional metacognitive beliefs [55]. The MSAS_18_ was administered as a measure of metacognitive functioning, evaluating five targeted metacognitive abilities, including monitoring, differentiation, integration, decentration, and mastery, on a 5-point frequency scale, with higher scores indicating better metacognition than low ones [56]. BIS_11_ was used to assess trait impulsiveness, measuring global, motor, non-planning, and attentional impulsivity on a 4-point frequency scale, with higher scores indicating greater impulsivity [57]. Total scores from the MSAS_18_, MCQ_30_ and BIS_11_ were included as psychological variables in the statistical analyses.

### 2.5. Experimental Design

**Figure 2:**
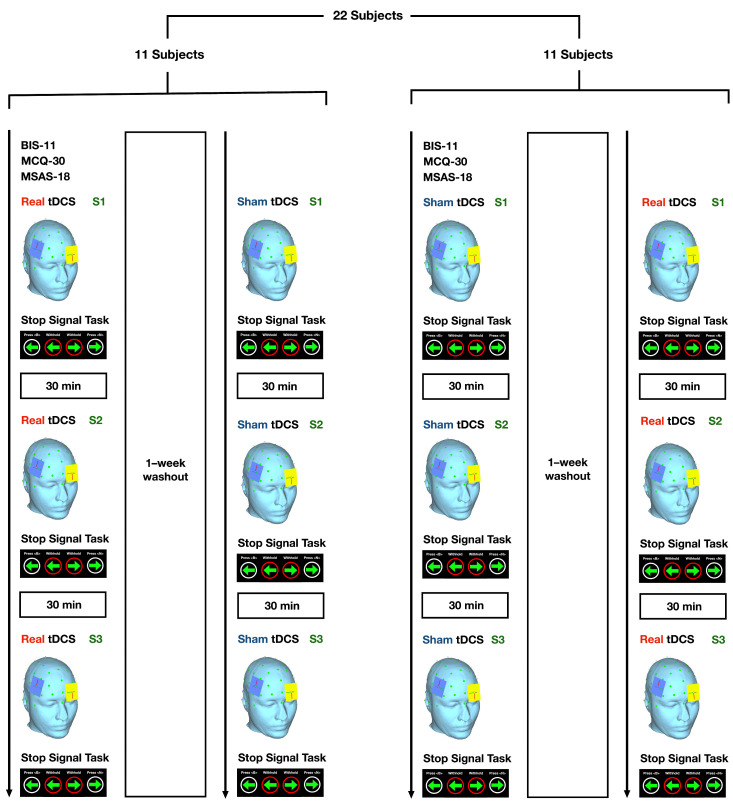
Experimental procedure. Each participant took part in two experimental sessions. At the start of each session, they completed the Metacognitions Questionnaire-30, the Metacognitive Self-Assessment Scale-18, and the Barratt Impulsiveness Scale-11. Subsequently, half of the participants underwent three sessions of real (active) tDCS, while the other half received three sessions of sham stimulation, with sessions spaced 30 min apart. During stimulation, all participants remained at rest. After each stimulation session, they completed the Stop-Signal Task. Following a 1-week washout period, participants returned to the laboratory to complete the Stop-Signal Task once more. This time, they received the stimulation protocol opposite to that administered in their previous sessions.

A double-blind, sham-controlled, within-subjects research design was adopted. Before stimulation, participants completed the MSAS_18_, MCQ_30_ and BIS_11_. Thereafter, they underwent three stimulation sessions within the same day (S1, S2, and S3), during which inhibitory-control performance was assessed via the SST. Importantly, the SST was administered after each stimulation block, once current delivery had ended, allowing repeated post-stimulation assessments across the accelerated protocol. The S1 and S2 sessions were separated by a 30-min rest period, and the same 30-min rest interval was maintained between the S2 and S3 sessions (see Bystad et al. [45] for a similar accelerated stimulation schedule). Such a research design was based on the same stimulation framework used in the reference paper [34], yet extended it by repeating the intervention for three times within a single day (see Figure 2 for a graphical representation).

### 2.6. Statistical Analysis

To conduct inhibitory control-related statistical analysis, Stop-Signal Reaction Time (SSRT), reflecting the latency of the stopping process, was computed using the integration method applied to data from the SST. Consistent with the independent horse-race model [58], the SSRT was calculated by integrating the reaction time distribution to find the point at which the integral equals the probability of responding, p(respond|signal), for a specific delay, and then subtracting the SSD from the finishing time. Higher SSRT indicates poorer response inhibition, whereas lower SSRT denotes better inhibitory control [59]. The mean error rate was also computed by averaging the proportion of incorrect responses in Go and Stop trials. Multiple linear mixed-effects (LME) models were then fitted to the data. Fixed and random effects were specified according to the hypotheses tested in Section 3. The LME models were followed by analysis of variance (ANOVA) to extract the exact *F*- and *p*-values linked to each factor. For outcomes reaching statistical significance, post hoc pairwise contrasts were conducted on the estimated marginal means, with *p*-values adjusted using the Holm method [60]. Kenward–Roger tests were used to compare the fitted models, while graphical inspection of Q–Q plots and histograms was employed to assess the normality of both dependent variables and model residuals. Since all effects were defined a priori, no correction for multiple comparisons was applied. A post hoc sensitivity power analysis was also conducted using G*Power (Version 3.1) for a repeated-measures within–between interaction (*n* = 22, conditions = 2, repeated measurements = 3, correlation among repeated measures = 0.5, nonsphericity correction ε = 1), yielding an estimated power of 0.85 to detect a medium effect size (*f* = 0.25), with α = 0.05 and β = 0.15, respectively. The remaining analyses were conducted using custom-made algorithms developed in R (Version 4.5.3; https://www.r-project.org/, accessed on 1 April 2026).

## 3. Results

To evaluate the significance of stimulation’s effect on inhibitory control across the three sessions of tDCS, two different LME models were fitted to the data, according to the following formulas (Wilkinson notation):

*Y* ∼ 1 + Protocol_order + tDCS × (Time + MSAS_18_ + MCQ_30_ + BIS_11_) + (1∣ID) (1)

**Figure 3:**
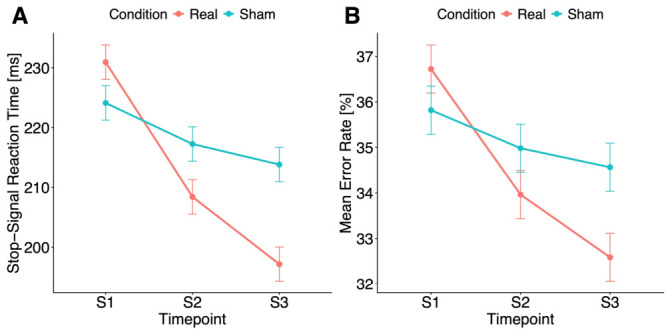
Stop-Signal Reaction Time and mean error rate across the three stimulation sessions. (**A**) Mean SSRT values are shown for the Real (red) and Sham (blue) conditions across the three timepoints (S1, S2, and S3). Error bars represent standard errors. The Real condition exhibited a marked decrease in SSRT compared to the Sham condition. (**B**) Mean error rate values are shown for the Real (red) and Sham (blue) conditions across the three timepoints (S1, S2, and S3). Error bars represent standard errors. No significant differences were detected between the Real and Sham conditions.

*Y* ∼ 1 + Protocol_order + tDCS × Time + (1∣ID) (2)

where *Y*, the response variable, was set as the SSRT or mean error rate. Among the fixed-effect factors evaluated, tDCS indicated the experimental condition (real or sham), Time reflected the effect of repeated assessment across the first day (S1, S2, S3), and Protocol_order indicated whether participants started with the real or the sham stimulation protocol. MSAS_18_, MCQ_30_, and BIS_11_ reflected subjects’ performance in the related questionnaires. Interaction terms were also added between tDCS and each questionnaire-derived variable. Finally, a random effect of subject was included. As far as SSRT is concerned, comparison of the two models using the Kenward–Roger test showed no significant difference (χ^2^(6) = 4.2430, *p* = 0.6438). Based on such a result, we performed the analyses on the more parsimonious model of Equation (2). ANOVA applied to the fitted model revealed a significant main effect of Time on SSRT (*F*(1, 107) = 17.6896, *p* < 0.0001, partial η^2^ = 0.142). Conversely, the main effects of tDCS (*F*(1, 107) = 1.7241, *p* = 0.1920, partial η^2^ = 0.016) and Protocol order (*F*(1, 20) = 0.0171, *p* = 0.8974, partial η^2^ = 0.001) were not statistically significant. Notably, a significant interaction was observed between tDCS and Time, (*F*(1, 107) = 4.2701, *p* = 0.0412, partial η^2^ = 0.038; see Figure 3A). Post hoc pairwise contrasts showed a significant difference between the real and sham conditions at S3, with lower SSRT values in the real condition (*t*(105) = 2.233, *p* = 0.0277, Cohen’s *d* = 0.436). Moreover, within the real stimulation condition, SSRT was significantly reduced from S1 to S2 (*t*(105) = 2.295, *p* = 0.0474, Cohen’s *d* = 0.448) and from S1 to S3 (*t*(105) = 4.207, *p* = 0.0002, Cohen’s *d* = 0.821), whereas the difference between S2 and S3 did not reach statistical significance (*t*(105) = 1.911, *p* = 0.0587, Cohen’s *d* = 0.373). In contrast, no significant effects were found in the full model for MSAS_18_, MCQ_30_, BIS_11_, or for their interactions with tDCS. Overall, these findings suggest that inhibitory-control performance progressively improved across the three stimulation sessions, indicating a gradual reduction in SSRT over time with repeated stimulation. See Table 1 for a summary of SSRT at each timepoint across the two stimulation conditions.

As far as error rate is concerned, comparison of the two models using the Kenward–Roger test showed no significant difference (χ^2^(6) = 5.0797, *p* = 0.5336). Therefore, subsequent analyses were conducted on the more parsimonious model of Equation (2). ANOVA applied to the fitted model revealed a significant main effect of Time (*F*(1, 107) = 6.8885, *p* = 0.0099, partial η^2^ = 0.060), yet no significant main effects of tDCS (*F*(1, 107) = 0.7263, *p* = 0.3960, partial η^2^ = 0.007) and Protocol order (*F*(1, 20) = 0.1754, *p* = 0.6798, partial η^2^ = 0.009) were detected on the mean error rate. Also, the interaction between tDCS and Time did not reach statistical significance (*F*(1, 107) = 1.6721, *p* = 0.1987, partial η^2^ = 0.015; see Figure 3B), indicating that the pattern of mean error rate did not change across sessions between the real and sham stimulation conditions. See Table 1 for a summary of mean error rate at each timepoint across the two stimulation conditions.

## 4. Discussion

The present study investigated whether an accelerated anodal tDCS protocol over the rIFG could induce cumulative improvements in inhibitory control that remain detectable in the short-term following each stimulation. Our findings showed a progressive reduction in SSRT across the three post-stimulation assessments, suggesting a cumulative, session-dependent enhancement of inhibitory control. However, this effect should be interpreted with caution given the marginal significance of the interaction effect and its small-to-medium effect size. This result is in line with our previous work [34], in which anodal stimulation of the rIFG improved inhibitory control, and it is consistent with earlier studies showing enhanced response inhibition following rIFG stimulation [29,30,31]. Theoretically, this pattern is also compatible with the established role of the rIFG within the distributed fronto-basal-ganglia network supporting response inhibition [9,10,11,12,13,14].

Compared with our previous study, the present findings further suggest that the effect is not merely observable during stimulation, but may also be detectable shortly after stimulation has ended and may accumulate across repeated administrations delivered within the same day. In this sense, the progressive reduction in SSRT across sessions may reflect a gradual facilitation of stopping performance induced by repeated modulation of rIFG excitability. This pattern must, however, be interpreted in terms of changes in performance trajectory across sessions rather than absolute performance levels. This interpretation is also coherent with the view that stimulation effects on inhibitory control are more readily captured by the Stop-Signal task than by the Go/NoGo task, since the literature has highlighted more robust effects for the former, whereas findings with the latter are generally less consistent [32,33,61,62]. Nonetheless, the absence of a pre-stimulation baseline on each experimental day limits the interpretation of early session differences and should be addressed in future studies.

Another relevant finding concerns the role of individual differences. In our previous study, stimulation effects were modulated by metacognitive beliefs, suggesting that neuromodulation outcomes may depend on the broader cognitive profile of the individual [34]. In the present study, however, we did not replicate the relationship between MCQ_30_ scores and stimulation effects. One possible explanation is that metacognitive factors may be particularly relevant when stimulation is acting online, that is, while participants are performing the task under direct modulation of cortical excitability. This possibility is coherent with the framework proposed in the original paper, according to which metacognition and inhibitory control are functionally related but their interaction with tDCS may be context-dependent [36,37,39,40,63].

Taken together, these findings confirm the relevance of the rIFG as a target for modulating inhibitory control. Although the observed cognitive enhancement is consistent with a stimulation-related facilitation beyond a general practice effect, the potential contribution of differential practice sensitivity cannot be completely ruled out and warrants further investigation in larger controlled studies. As no additional delayed assessment was collected later on the same day after the stimulation, future studies should also aim to determine whether the effects of the present tDCS protocol remain stable over time (i.e., across several hours or days). In this regard, it will be important to investigate whether more prolonged interventions, repeated stimulation across multiple days, or combined approaches integrating neuromodulation with cognitive or metacognitive interventions can lead to stable effects over time. Previous investigations suggest that combining repeated prefrontal tDCS sessions with cognitive training may enhance response inhibition more effectively than training alone [49,64], while metacognitive psychotherapy may represent a useful adjunct for maximizing the effects of neuromodulation on higher-order cognitive functions [65].

Future studies should further refine this line of research by combining neuromodulation with direct measures of large-scale circuit dynamics. In particular, it may be useful to pair stimulation protocols, not only anodal tDCS, but also other approaches such as alternating-current stimulation, with connectomic measures capable of capturing the functional organization of inhibitory-control networks. A specific focus on functional connectivity could help clarify the neural substrates underlying the behavioral effects observed here and determine whether repeated stimulation induces measurable changes in the communication among prefrontal and subcortical regions involved in response inhibition. Such an approach may be especially valuable for understanding why behavioral improvements appear to accumulate during stimulation. Supporting this perspective, long-term evidence from neurological patients suggests that combining electroencephalography-based profiling with electrical stimulation induces sustained neuroplastic changes and promotes motor and functional recovery beyond that achieved with conventional rehabilitation protocols [66].

In addition, the present study was conducted in healthy participants, and future work should investigate whether similar protocols may prove more informative or clinically relevant among patient populations characterized by inhibitory-control deficits. In particular, these studies could establish optimal dosing parameters, include larger follow-up windows (i.e., 3-month and 6-month assessments), and consider individualized electrode placement or individualized stimulation targeting via neuroimaging-guided approaches or functional connectivity mapping. Extending this paradigm to clinical groups could help determine whether neuromodulatory interventions targeting inhibitory-control networks may have greater translational value in populations for whom this function is more markedly compromised.

## Figures and Tables

**Table 1:** Summary of fitted SSRT and mean error rate (±standard error) at S1, S2, and S3 for real and sham stimulation conditions.

		tDCS Protocol	
Response Variable	Time	Real (*n* = 22)	Sham (*n* = 22)
Stop-Signal Reaction Time	S1	230.95 ± 2.88	222.53 ± 2.88
	S2	208.43 ± 2.88	217.27 ± 2.88
	S3	197.18 ± 2.88	213.83 ± 2.88
Mean Error Rate	S1	36.72 ± 0.53	35.82 ± 0.53
	S2	33.97 ± 0.53	34.98 ± 0.53
	S3	32.59 ± 0.53	34.57 ± 0.53

## Data Availability

The raw data supporting the conclusions of this article will be made available by the authors on request.

## Abbreviations

The following abbreviations are used in this manuscript:

rIFG	right Inferior Frontal Gyrus
NIBS	Non-Invasive Brain Stimulation
tDCS	transcranial Direct Current Stimulation
EEG	Electroencephalography
SST	Stop-Signal Task
SSD	Stop Signal Delay
MCQ_30_	Metacognitions Questionnaire-30
MSAS_18_	Metacognitive Self-Assessment Scale-18
BIS_11_	Barratt Impulsiveness Scale-11
SSRT	Stop-Signal Reaction Time
LME	Linear Mixed-Effects (models)
ANOVA	ANalysis Of VAriance
